# Society Proceedings

**Published:** 1903-02

**Authors:** 


### SOCIETY PROCEEDINGS.

### PASSAIC COUNTY VETERINARY MEDICAL ASSOCIATION.

The regular monthly meeting of the Passaic County Veterinary Medical Association was held at 169 Paterson Street, Paterson, N. J., on Tuesday evening, January 6, 1903, with Dr. William Herbert Lowe, President, in the chair, and Dr. T. J. Cooper acting as Secretary.

On roll-call the following members answered to their names: Drs. William J. Fredericks, Delawanna; T. J. Cooper, Paterson; John H. De Graw, Paterson; J. Payne Lowe, Passaic; W. H. Lowe, Jr., Paterson, and William Herbert Lowe, Paterson.

The minutes of December 2d were read and approved.

### New Business.

Dr. Cooper brought up the matter of arranging to provide a substitute in the event of a practitioner being sick or called out of town.

Dr. Cooper thought one of the best ways of promoting the mutual interests of members was by each practitioner reporting from time to time such cases in his practice as were not responding satisfactorily to treatment.

Dr. J. Payne Lowe was in favor of the Association paying the expenses of practitioners in sending specimens to the State Laboratory at Trenton for bacteriological examination in cases where the Association received the benefit of such examination. He cited cases of dumb rabies in the dog, diphtheria in the cat, and so on, where the practitioner from a clinical standpoint readily made out a diagnosis, but where in many instances it would be advisable to have the diagnosis confirmed by bacteriological examination or inoculation experiments.

The several matters brought up under new business were laid over.

A letter was read from Dr. William C. Berry to the effect that he was quarantined at his home in the upper part of the county on account of the existence of scarlet fever in his family, and consequently could not attend.

On motion of Dr. W. H. Lowe, Jr., the Secretary was instructed to write a suitable letter to Dr. Berry conveying to him the sympathy of his fellow members.

Dr. Fredericks read a paper giving his experiments with iodide of potassium and eserine in the treatment of parturient paresis. Three cases that he had treated with these drugs recovered. Dr. Fredericks injects 3 grains eserine subcutaneously. Dr. J. Payne Lowe remarked that there was one thing sure, and that was that we did not kill any of our cases of parturient paresis now by drenching since we had adopted the modern method of treatment.

Dr. Cooper told of a peculiar case of lameness in a mare in foal, and promised to produce the mare in evidence at the next meeting.

The President appointed Dr. W. H. Lowe, Jr., essayist for the next meeting.

On motion, meeting adjourned at 10 P. M.

T. J. Cooper,
Secretary *pro tem.*

### ANNUAL MEETING OF THE VETERINARY MEDICAL ASSOCIATION OF NEW JERSEY.

The annual meeting of the Veterinary Medical Association of New Jersey was called to order on the morning of January 8, 1903, in the parlors of the Trenton House, Trenton, N. J. The President, Dr. William Herbert Lowe, occupied the chair. Records of the semi-annual meeting, held at Newark, July 10, 1902, were read and approved.

Applications for membership were on file, as follows:

Dr. Vernon B. Height, of Asbury Park; Dr. H. R. Clark, of Long Branch; Dr. J. H. Conover, of Flemington; Dr. J. B. Jones, of Atlantic City.

All applicants were duly vouched for and approved by the Executive Committee, and were by unanimous vote admitted to membership.

A bill to change Article III. of the Constitution came before the members for final action. This bill, which came before the last semi-annual meeting for first reading, had bearing upon eligibility for membership. The change proposed was essential owing to the existence of the new law enacted at the last session of the Legislature, and provided that candidates for membership entering the profession on or after the first Monday in May, 1902, must be licensed by the State Board of Veterinary Medical Examiners and be registered in conformity with the provisions of chapter 18, laws of 1902. By vote of the Association the change in the Constitution was adopted.

A report from the Legislation Committee revealed the fact that there were some violations of the newly-enacted law regulating the practice of veterinary medicine, surgery, etc. Dr. A. T. Sellers, of Camden, reported a case of illegal practice. Dr. Vernon B. Height, of Asbury Park, reported that with Dr. H. R. Clark, of Long Branch, he had been instrumental in the arrest of an illegal practitioner in his section of the State. The case was to have come before the Grand Jury on the day of the meeting, January 8th, and the fact that the defendant had communicated with his accusers, seeking for clemency, would indicate his acknowledgment of guilt and fear of retribution.

The report of the Legislation Committee brought forth some discussion as to the proper method of dealing with violators of the law.

Drs. T. E. Smith and T. B. Rogers, of the State Board of Examiners, spoke for the Board, and requested that cases of violation of the law be reported to them whenever the evidence was of such a character as to warrant prosecution.

The special committee appointed to investigate delinquents reported that letters had been sent to or personal calls had been made upon such members, and that in some cases no reply had been received from courteous communications; that in some other cases, where a personal visit was made, members of the committee had been treated in a discourteous and insulting manner. It was recommended that eleven names be dropped from the Association membership.

The vote of the Association to act in accordance with the recommendation of the committee was a unanimous one.

The following delegates reported:

Dr. T. Earle Budd for the delegation to the Atlantic City Horse Show.

Drs. Smith and Glennon, delegates to the recent meeting of the American Veterinary Medical Association at Minneapolis.

Dr. L. P. Hurley, delegate to the Pennsylvania Veterinary Medical Association, and Dr. S. S. Treadwell, to the New York State Association.

At this time, under the regular order of business, occurred the election of officers for the ensuing two years. The following were unanimously chosen by ballot:

President, Dr. William Herbert Lowe, Paterson; First Vice-President, Dr. T. B. Rogers, Woodbury; Second Vice-President, Dr. H. Vander Roest, Newark; Secretary, Dr. George W. Pope, Athenia; Treasurer, Dr. James M. Mecray, Maple Shade.

It was voted that $50 per annum be devoted to the Secretary's use for employing a stenographer and typewriter.

Following the election of officers, the President made his annual address. It is probable that this address will be published in the near future, so but passing mention will be made of the chief suggestions, which were:

1. The appointment of a conference committee to confer with State officials, visit State agricultural and experiment stations, etc.
2. The establishment of a State veterinary library.
3. A two days' session of the Association.
4. The establishment of a bureau with a State veterinarian in charge, said bureau to be to the State what the Bureau of Animal Industry is to the country at large.

Following the President's address the following visitors were introduced:

Dr. E. B. Voorhees, President of the State Board of Agriculture; Hon. Franklin Dye, Secretary of the State Board of Agriculture; Hon. S. B. Kitcham, of the State Tuberculosis Commission; Dr. Veranus A. Moore, of Ithaca, N. Y.; Drs. Bell, Berns and Ackerman, of Brooklyn; Robertson, Ellis, and Dixon, of New York; Pearson and Hoskins, of Philadelphia.

Dr. Voorhees highly commended the suggestions embodied in the President's address, and explained the need of some one division of the State government to which animal industry matters could be referred. At the present time there is existing the State Board of Agriculture, the Tuberculosis Commission, and the Dairy Commission, to which bodies communications and requests are frequently sent indiscriminately, thus necessitating delay and question as to the extent of authority of these several divisions of the public service.

Hon. Franklin Dye, Secretary of the State Board of Agriculture, also addressed the meeting and invited the Association to apply for membership in the State Board of Agriculture.

It was voted to make such application, and if received, empower the President to appoint the two delegates allowable to the Association as members.

At 1 P.M. the meeting adjourned for dinner, reconvening at 2.30.

Under new business, Dr. H. Vander Roest stated that the Essex County Board of Health would without doubt in the near future appoint a meat-inspector, and it was voted that the Secretary be authorized to communicate with the Board and respectfully request that in case such an appointment was made, the office be filled by a qualified veterinarian.

It was also voted that a conference committee be appointed with power to carry out the suggestions incorporated in Dr. Lowe's address. The literary feature of the meeting was a paper presented by Dr. Veranus A. Moore, of Cornell University. The paper was entitled "Etiology and Prevention of Infectious Diseases of Animals."

Following the reading of the paper, Dr. Moore, with the aid of lantern slides, gave a fine demonstration of various forms of bacteria. It would not be possible to reproduce Dr. Moore's extemporaneous remarks while the stereopticon was in operation, but suffice it to say that he held the attention of his hearers from first to last. Dr. Moore's capital paper will, without doubt, appear in the columns of our veterinary magazines.

Dr. T. B. Rogers, of the State Board of Examiners, read a paper, entitled "The Relation of the State Boards of Examiners to the Teaching Schools, the Profession and the State." Dr. Rogers' paper elicited a discussion, in which Drs. Robertson, Hoskins, Berns, Pearson, Ackerman, Bell, and others participated.

Dr. Pearson made a brief report of his experiments in immunizing animals against tuberculosis.

It was voted to hold the semi-annual meeting in July, 1903, with the Secretary at Athenia.

President William Herbert Lowe announced the appointment of committees and delegates as follows:

*Executive Committee.*

Dr. T. Earle Budd, *Chairman*, Orange; Dr. J. O. George, Camden; Dr. E. L. Loblein, New Brunswick; Dr. J. B. Hopper, Ridgewood, and Dr. L. P. Hurley, Hopewell.

*Public Health Committee.*

Dr. Werner Runge, *Chairman*, Newark; Dr. J. V. Laddey, Arlington; Dr. Thomas H. Ripley, Newark; Dr. Edwin Rowe, Jr., Summit, and Dr. W. F. Harrison, Bloomfield.

*Animal Industry Committee.*

Dr. J. Payne Lowe, *Chairman*, Passaic; Dr. D. J. Dixon, Hoboken; Dr. J. B. Finch, Ramseys; Dr. James McDonough, Montclair, and Dr. W. J. Fredericks, Delawanna.

*Legislation Committee.*

Dr. William Herbert Lowe, *Chairman*, Paterson; Dr. Henry Vander Roest, Newark; Dr. Charles T. Magill, Haddonfield; Dr. T. E. Smith, Jersey City; Dr. Whitfield Gray, Newton, and Dr. R. T. Churchill, Secaucus.

*Finance Committee.*

Dr. James T. Glennon, *Chairman*, Newark; Dr. Wm. Gall, Matawan, and Dr. J. H. Conover, Flemington.

*Publication Committee.*

Dr. J. O. George, *Chairman*, Camden; Dr. E. Matthews, Jersey City, and Dr. R. O. Hasbrouck, Passaic.

*Press Committee.*

Dr. T. Earle Budd, *Chairman*, Orange; Dr. William Dimond, Newark, and Dr. T. B. Rogers, Woodbury.

*Conference Committee.*

Dr. William Herbert Lowe, *Chairman,* Paterson; Dr. T. B. Rogers, Woodbury, and Dr. James H. Mecray, Maple Shade.

*Committee on Arrangements* (Athenia Meeting).

Dr. George W. Pope, *Chairman,* Athenia; Dr. J. Payne Lowe, Passaic, and Dr. Werner Runge, Newark.

*Representatives on the State Board of Agriculture.*

Dr. George W. Pope, Athenia, and Dr. George F. Harker, Trenton.

*Delegates to the New York State Veterinary Medical Society.*

Dr. R. F. Meiners, Rutherford; Dr. S. S. Treadwell, Englewood; Dr. George E. Fetter, Hopewell; Dr. J. M. Everett, Hackettstown, and Dr. William Gall, Matawan.

*Delegates to the Pennsylvania State Veterinary Medical Association.*

Dr. George C. Forsyth, Columbus; Dr. H. R. Clark, Long Branch; Dr. Vernon B. Height, Asbury Park; Dr. G. Walter Dilkes, Mullica Hill, and Dr. H. W. Read, Freehold.

*Delegates to the New Jersey Sanitary Association.*

Dr. J. V. Laddey, Arlington; Dr. Edwin Rowe, Jr., Summit, and Dr. L. E. Tuttle, Bernardsville.

*Delegates to the American Veterinary Medical Association.*

Dr. T. E. Smith, Jersey City; Dr. John P. Matthews, Princeton; Dr. R. J. Halliday, Bayonne; Dr. E. A. Hogan, Newark; Dr. James Mosedale, Morristown.

*Delegates to the Atlantic City Horse Show.*

Dr. T. Earle Budd, Orange; Dr. J. B. Jones, Atlantic City; Dr. William P. Fink, Atlantic City.

GEORGE W. POPE,
Secretary.

---

### ALLEGHENY COUNTY VETERINARY MEDICAL ASSOCIATION.

Vice-President Rectenwald presided over a large and enthusiastic meeting, held at the office of Dr. J. E. Spindler, on the evening of January 28th.

Members present: Drs. Ainsworth, Boyd, Gearhart, Gilmore, McNeil, Rectenwald, Richards, Spindler, Spohn, Taylor, Waugh. Visitors: Drs. Bittles and Porter, of New Castle; Hoskins, of Philadelphia; Laberge, of Beaver Falls; Jones, of Pittsburg; Magee, of Uniontown; Prothero, of Johnstown; W. J. Waugh, of Washington, and Weitzel, of Allegheny, Pa.

Dr. George Magee read a well-prepared paper on "Animal Life in the Coal Mines," especially those stabled in shaft coal mines. Discussion by Drs. Rectenwald, McNeil, W. J. Waugh, Weitzel, Laberge, and J. A. Waugh.

Dr. W. B. Prothero reported an operation in trifacial neurectomy, followed by much swelling and irritation of the lips and lower part of the head, which did not yield readily to treatment, but appeared to abate or recover spontaneously on the fourth day after the operation. This is the Prof. W. L. Williams' operation for involuntary head-shaking, and proved successful; and the patient remained under observation about fifteen months. This subject proved so interesting that many questions were asked and answered, and nearly everyone present took part in the discussion.

Dr. C. Z. Laberge reported having treated twenty-eight cases of tetanus in horses with Mulford's antitoxin, and having lost no cases since using it. This is a remarkable clinical experience, illustrating the value of serum treatment.

Dr. N. Rectenwald made some practical remarks on obstetrics and modern surgery; exhibited his obstetric outfit, and showed some recently perfected instruments.

Dr. D. C. Gearhart made a stirring address on the prosecution of illegal practitioners, and incidentally made some strenuous remarks on our duties to ourselves and fellow-practitioners, with charity toward the poor, and prompt collection of fair-sized bills from the rich. Friendly discussion prevailed.

Dr. W. Horace Hoskins made one of his characteristic addresses on the "Practitioner, the Public, and the Veterinary Profession in Their Various Relations."

A spirit of good-fellowship and conversation prevailed, and the Association was reorganized, enlarged, and will be perfected at a special meeting on February 11th.

JAMES A. WAUGH, V.S.,
Secretary.

---

### THE ILLINOIS VETERINARY MEDICAL AND SURGICAL ASSOCIATION.

THIS Association met in annual session at the St. Nicholas Hotel, Decatur, Ill., January 14, 15, 1903. The meeting was called to order by President Dr. V. G. Hunt, of Arcola, Ill. Following the roll-call, which was responded to by a goodly number of the membership, President Hunt delivered an exceedingly interesting and very instructive address, which was warmly applauded, and was as follows:

Gentlemen: As we meet to-day, we may well rejoice at the rapid strides made by the profession within the past few years. The profession no longer pins its faith to theories that cannot be demonstrated. Nothing but absolute fact will satisfy. Every veterinarian is doing some original thinking, and does not rush into print with a new discovery until it has been demonstrated to be practical. Drugs have become so cheap that the veterinarian can give our domestic animals the benefit of all the new and best remedies with comparatively small cost. What an advantage the young man of to-day has over the old practitioner who gained his knowledge thirty years ago! While he was in practice he had to be a diligent student to keep up with the times. Necessity kept him on the alert, but to-day the novice has his text-books, his lectures, and, last but not least, his veterinary journal, and if Dame Nature has been liberal with brains he will make a success.

We see by our dailies that in the East that terrible foot-and-mouth disease has invaded their territory. The veterinarians are already on the ground, and we trust will stamp it out. The utmost precaution will always be necessary from the fact that, to improve our flocks and herds, importations from the infected districts must be made in spite of the danger. The veterinarian must be on the alert; not to pose as an alarmist, but, where the symptoms are imperfectly developed, use every precaution to insure the safety of the herd, even if his worst fears were never realized. The veterinarian of to-day has diseases to contend with that were little known thirty years ago, some of which are of such a malignant character that they must be met in their incipiency or a fearful loss will be the result. Most liberal appropriations will have to be made to meet the necessary expense. Conditions are constantly changing that will require nice diplomacy to not inflict a hardship on unfortunate owners in infected districts. Then the veterinarian with practical experience, coupled with a clear head, will do his duty without fear, favor or affection.

All Europe is watching our live-stock interests with a jealous eye, and will look with no degree of composure, which will require us to be alive to our individual interests. Our exports to other climes are immense. We can hardly conjecture what they will be within the next decade. Let us hope we may meet every emergency, so American interests will be subserved.

Our late veterinary law without doubt has not met the expectations of its authors, unless the fee was the focus of their thoughts and the height of their ambition. Be this as it may, in many localities quackery is as prevalent as ever. Let us hope in the near future the stock-owner himself, unaided by State legislation, will give each practitioner his proper place. Individual interest is a wonderful safeguard. True merit will eventually win. Glittering theories are very inspiring, but avail little in the sick stall. Here is where the true veterinarian shows his skill. We trust our beloved profession in the near future will reach such perfection that our domestic animals will be treated on the same intelligent basis as their lordly master. We owe this to that all-wise Creator who placed them in our care.

When sick do not fly to some therapeutic arsenal for a remedy without any thought as to their ailments. Is it to be wondered at if he loses his valuable animal under these circumstances? Is it any wonder the public becomes disgusted at the impotency of the healing art? One writer has truly said, "Medicine in incompetent hands is like a sword in the hands of a madman."

The world is full of vain pretenders with a hemorrhage of words, gaudily dressed, and, for a time, capable of deceiving the very elect. This forcibly reminds us of a $40 bill on a $20 horse; but all they have to do is to move a little farther along and catch another sucker. Thus it has been and will be to the end of time.

The conscientious veterinarian realizes his ability, and never dreams of trickery; is strictly honest with his patrons, and enjoys their well-merited confidence.

Now, in conclusion, let me most heartily thank you for your most valuable help while President of your Society, and in retiring will hold every member in grateful remembrance, hoping their usefulness will increase with their years, hoping and trusting my successor will receive the same valuable support so generously extended to me. May the Society be inspired with new life by a younger man. Our Society has a world-wide reputation, capable of doing much good, located in one of the richest agricultural districts on the globe. Its inhabitants represent every civilized country on earth, and the very best of them. Need we fail under such circumstances?

The minutes of the previous meeting were then read and approved by the Association.

The election of officers for the ensuing year followed, and resulted as follows: President, Dr. V. G. Hunt, Arcola, Ill.; Vice-Presidents, Dr. A. Travis, Litchfield, Ill., and Dr. F. D. Bliss, Earlville, Ill.; Secretary, Dr. W. A. Swain, Mt. Pulaski, Ill.; Treasurer, Dr. J. M. Reed, Mattoon, Ill.

Three new members—Dr. H. P. McKinney, Neoga, Ill.; Dr. C. F. Griffin, Newman, Ill., and Dr. F. D. Bliss, Earlville, Ill.—were duly elected after passing the requisite examination, and were introduced to the members by the Secretary.

STANDING COMMITTEES APPOINTED BY THE PRESIDENT:

*Committee on Membership*—Drs. S. H. Swain, J. M. Reed, A. Travis, C. A. Hurlbutt, and W. C. Dawson.

*Committee on Program*—Drs. V. G. Hunt, W. A. Swain, and S. H. Swain.

*Committee on Arrangements*—Drs. V. G. Hunt and F. Glassbrenner.

*Committee on Legislation*—Drs. John Osborne, V. G. Hunt, S. H. Swain, and C. A. Hurlbutt.

The following papers were read: "Retained Fœtal Envelopes," by Dr. J. M. Reed, of Mattoon, Ill., which was very instructive and interesting, and was responded to by Drs. V. G. Hunt and S. H. Swain. "Œsophageal Obstruction," by Dr. C. A. Hurlbutt, of Stonington, Ill., was treated in a most creditable manner. "Persistency of the Urachus" being next on the program, Dr. S. H. Swain, of Decatur, Ill., read a very concise and instructive paper on the same, which was responded to by Drs. J. W. Marsh and C. A. Hurlbutt. Dr. John Osborne next read a most entertaining paper on the subject of "Vegetable Poisoning Milk Sickness," which was responded to by Dr. V. G. Hunt and others. The subject of "Parasitic Bronchitis" was next treated by Dr. J. W. Marsh, of Illiopolis, Ill., in a very interesting and instructive manner. He was responded to by Drs. S. H. Swain and F. J. Bliss.

Under the head of "Report of Cases," Dr. A. Travis, of Litchfield, Ill., read a report of an operation for "Scrotal Hernia," which was well received and discussed at length.

Dr. W. A. Swain, of Mt. Pulaski, Ill., reported a case of "Fungosis Toxicum Paralyticus" in his practice which was of more than passing interest, and brought forth some animated discussion. "Cerebro-spinal Meningitis," by Dr. V. G. Hunt, and many other extremely interesting subjects were discussed.

Among the visitors present were Drs. Ansell Gould, Bone Gap, Ill.; J. B. Edgar, Ivesdale, Ill.; A. Robertson, Mt. Carmel, Ill.; John Turrell, Mackinaw, Ill.; Charles E. Howard, Lebanon, Ill.; P. L. Stombaugh, Altamont, Ill., and W. Smith, Tinley, Ill.

The date and place of the next meeting was fixed for August 12 and 13, 1903, at Decatur, Ill.

Meeting adjourned until the above date. DR. W. A. SWAIN,
Secretary.

